# The role of experiential knowledge within attitudes towards genetic carrier screening: A comparison of people with and without experience of spinal muscular atrophy

**DOI:** 10.1111/hex.12602

**Published:** 2017-07-13

**Authors:** Felicity K. Boardman, Philip J. Young, Oliver Warren, Frances E. Griffiths

**Affiliations:** ^1^ Division of Health Sciences Warwick Medical School University of Warwick Coventry UK; ^2^ School of Life Sciences University of Warwick Coventry UK

**Keywords:** ethics, experiential knowledge, genetic screening, spinal muscular atrophy

## Abstract

**Purpose:**

Autosomal recessive conditions, while individually rare, are a significant health burden with limited treatment options. Population carrier screening has been suggested as a means of tackling them. Little is known, however, about the attitudes of the general public towards such carrier screening and still less about the views of people living with candidate genetic diseases. Here, we focus on the role that such experience has on screening attitudes by comparing views towards screening of people with and without prior experience of the monogenetic disorder, Spinal Muscular Atrophy.

**Methods:**

An exploratory sequential mixed methods design was adopted. In‐depth qualitative interviews were used to develop two surveys. The surveys addressed attitudes towards carrier screening (pre‐conceptual and prenatal) for SMA.

**Participants:**

337 participants with SMA experience completed the SMA Screening Survey (UK) and 336 participants with no prior experience of SMA completed the UK GenPop Survey, an amended version of the SMA Screening Survey (UK)**.**

**Results:**

The majority of both cohorts were in favour of pre‐conception and prenatal carrier screening, however people with experience of type II SMA were least likely to support either. Key differences emerged around perceptions of SMA, with those without SMA experience taking a dimmer view of the condition than those with.

**Conclusion:**

This study underscores the significance of prior experience with the condition to screening attitudes. It highlights the need for accurate and high‐quality educational resources to support any future carrier screening programmes, that particularly in relation to rare genetic disorders like SMA that will fall outside the remit of everyday experience for the majority of the population.

## 1 INTRODUCTION

As reproductive genetic medicine advances and the number of conditions that can be detected through the use of genetic technologies increases, important questions need to be addressed about which conditions are suitable candidates for expanded genetic screening. While currently in the UK, NHS genetic testing is typically reserved for families with a known history of (or identified risk factors for) the particular genetic disorder in question, the development of technologies such as whole genome sequencing (WGS) are beginning to alter the landscape and remit of genetic screening practices. Indeed, WGS allows for large panels (50+ conditions) of genetic conditions to be screened for simultaneously,^1^ making screening on a mass scale more technologically, practically and financially feasible than ever before. Furthermore, WGS is already expanding in its role within mainstream NHS health care, particularly in the areas of diagnostics and reproduction^2^, ^3^ bringing to the fore questions of whether and how such large volume screens could (or indeed *should*) be offered to the general population with no known risk factors for genetic disease.^4^

One of the key arguments in favour of the expansion of genetic carrier screening, particularly pre‐conception genetic screening (PCGS), concerns its capacity to extend and enhance the reproductive options of would‐be parents who are carriers of genetic disorders.^5^ Currently, such parents most commonly learn of their genetic carrier status after the birth of an affected child. PCGS, however, by alerting prospective parents to their carrier status before a pregnancy is even conceived, increases the reproductive options of such couples by permitting them access to reproductive technologies (eg pre‐implantation genetic diagnosis, the use of donated gametes) for use in their *first,* rather than subsequent, pregnancies. Prenatal genetic screening (PNGS) on the other hand, through carrier screening of pregnant women, their partners and/or foetuses would give expectant carrier parents the (otherwise inaccessible) option of preventing the birth of an affected foetus altogether (through selective pregnancy termination), or provide them with the information required to prepare for the significant life changes that typically accompany the birth of a child with a serious genetic disorder.

In spite of the potential for increased reproductive autonomy afforded by the use of genetic screening, as outlined above, however, its expansion would also represent a significant shift in the nature and type of reprogenetic decisions facing would‐be parents. Carrier screening would demand that such parents make life‐altering reproductive decisions based on the abstract notion of their genetic carrier status rather than the more tangible experience of genetic disease in their family.^6^ Indeed, they may be required to make screening decisions in relation to conditions they are likely to have never come across, or even heard of before. As such, reproductive decision‐making appears set to be increasingly dislocated from the experiential reality of genetic disease.

While various studies have been carried out to gauge public attitudes towards the expansion of genetic carrier screening,^1^ far fewer have considered the views of families currently living with genetic diseases.^7^, ^8^ Such families can be described as possessing direct “experiential knowledge” of the condition in question.^9^ This experiential knowledge has variously been described as “embodied” (ie having the condition oneself), or “empathetic” (ie experiencing the condition through close proximity to a person with it). Both of these forms of knowledge have been demonstrated to play a key role in shaping and informing the reproductive attitudes and decisions of families living with genetic diseases in various different ways.^7^, ^8^, ^10^, ^11^, ^12^ Members of the general public, however, approach screening decisions from an entirely different vantage point comparatively to make the very same decisions which, up until now, were only made experienced affected families face: whether or not to use genetic technologies to prevent or avoid the birth of a child with that condition.^13^

In spite of this emerging body of literature, however, researchers exploring the acceptability, or potential impact, of genetic carrier screening have tended to focus their attention exclusively on either the reproductive attitudes of the general public^1^, ^14^, ^15^ (the targets of carrier screening) or (less often) the views of affected families.^16^ However, there remains very little cross‐referencing of this literature.^17^ This is in spite of the fact that such a comparison brings sharply into focus the disparity of knowledge, experience and information between affected families, and the people who (through carrier screening) will become the new generation reprogenetic decision‐makers in relation to that condition, the general public. Here, we report on such a comparison, to highlight the broader social and ethical concerns around expanded carrier screening, using Spinal Muscular Atrophy (SMA) as an example.

### 1.1 Spinal muscular atrophy

Spinal muscular atrophy (SMA) is the most common genetic cause of infant death worldwide and yet is generally considered to be a relatively obscure condition.^18^ It is an autosomal recessive disorder caused by deletions of the *Survival Motor Neuron* (*SMN*) gene.^19^, ^20^ SMN deletions trigger in the apoptotic loss of the alpha motor neurones of the spinal cord, result in progressive and symmetrical atrophy of the voluntary muscles of the limbs and trunk.

Spinal muscular atrophy includes clinical categories that are based on the severity of the disease and the age of onset.^18^ Type I SMA (severe SMA) emerges within the first 4 months of life, with affected children usually dying through respiratory failure within 2 years.^18^ Type II SMA (intermediate) is the most heterogeneous form, with onset usually within the first 2 years of life and most affected individuals living normal or near‐normal lifespans.^18^ Type III SMA is usually diagnosed after the age of 4 years, with the majority of able to sit and stand unaided (although this may later be lost) and life span usually unaffected.^18^ Type IV SMA is the form of SMA with the latest onset (typically in the 2^nd^ or 3^rd^ decade of life). Lifespan is unaffected in type IV SMA, although, like type III, adults with this form of SMA experience increasing level muscle weakness over time and most eventually lose the ability to walk unaided.

While the typing system for SMA has long been used as a shorthand for disease severity both within and without the medical profession, it is also acknowledged that this way of categorizing SMA results in types with a high degree of overlap between them and broad ranges of disease severity within them.^21^

### 1.2 Spinal muscular atrophy and genetic screening

As SMA is an autosomal recessive condition (a single gene disorder requiring two carrier parents to transmit), this means that each pregnancy conceived by carrier parents has a 1 in 4 chance of having SMA. Estimates of carrier frequency in the general population vary considerably, although generally fall within the region of 1:40 and 1:60. The condition is estimated to affect 1 in every 6000‐10 000 births worldwide.^22^ While prenatal testing and/or cascade carrier screening is routinely offered to families with a known history of SMA, screening of the general population is somewhat more controversial.^8^, ^23^, ^24^ Indeed, there is a distinct lack of consensus amongst leading authorities on whether screening is advisable.^25^, ^26^ Key concerns that have prevented a screening programme for SMA being introduced in the UK context include a lack of effective treatments, an accepted requirement for newborn screening^25^ (although the recent FDA approval of Nusinersen,^27^ a drug which has been demonstrated to improve the muscle function of some children with SMA, may lead to increased pressure to revise this policy), and the inability of screening tests to accurately distinguish disease severity.^25^ Data presented here is aimed at addressing whether this second concern is seen as a significant issue in both affected families and the general population.

Using the SMA Screening Survey (UK), we have previously reported that within a sample of 337 affected family members and adults with SMA, there was relatively widespread support for both PCGS and PNGS, although adults and families affected by type II SMA expressed the most resistance.^8^ Here, we compare and contrast the reproductive views and attitudes of these affected families to those of people in the general population. The presented data allows a thorough and detailed investigation of the current views on SMA screening in the UK and allows interesting insightful comparisons between different subpopulations of people set to be directly affected (albeit in contrasting ways) if screening for SMA were to be introduced. The general relevance to other potential screening programmes in the UK will also be discussed.

## 2 METHODS

An exploratory sequential mixed methods research design was adopted for this study. This design was chosen as it allows for both an in‐depth exploration of the complex and sensitive topic of screening, while also permitting the breadth and generalizability of analysis most commonly associated with quantitative approaches. The research took place in three distinct phases, involving qualitative interviews (phase I) and two surveys (phase II), before a merged quantitative analysis (phase III). This paper focuses on phases II and III of the study, with phase I qualitative data presented elsewhere.^7^, ^8^ The project was supported and guided by three expert panels of professionals involved with, and people living with, SMA. Ethical approval for the study was granted by the University of Warwick's Biomedical and Scientific Research Ethics Committee in July 2014.

### 2.1 Qualitative interviews: Phase I

To gain a deeper understanding of the nature of attitudes towards screening for SMA amongst affected families, in‐depth qualitative interviews were conducted with 36 people who either have SMA themselves, or have SMA in their family, recruited through the main support and advocacy group for people living with SMA in the UK, SMA Support UK. The interviews were designed to explore participants’ experiences with SMA, their views around, and experiences of, genetic testing technologies and selective termination, as well as their perceptions of PCGS/PNGS for SMA.

### 2.2 The SMA screening survey (UK): Phase II

Details of the development SMA Screening Survey (UK) have been presented in greater detail elsewhere.^8^ In brief, the survey was directly informed by phase I qualitative data. As the aim of this research programme was to explore the nature and spread of attitudes amongst families living with SMA key themes from phase I were initially used to delineate the key domains of the survey. These broad domains were then later transformed into single sentence “attitude/belief” statements, and subsequently developed into quantitative survey questions through the use of a Lickert scale. Using this approach, respondents were able to state their degree of agreement/disagreement with them. Where possible, the actual words of participants who articulately encapsulated a particular theme within their interview were used directly as an attitude statement.

Given that participants in the SMA Screening Survey (UK) were already aware of their genetic risk status (or the possibility of it) and therefore would not be the intended recipients of population‐level PCGS or PNGS, the survey was designed to capture their attitudes towards, rather than intended use of, these screening programmes. This was measured by the degree to which participants stated that they would support a PCGS or PNGS programme. Further questions were then developed to explore key reasons for support and non‐support.

### 2.3 UK GenPop survey: Phase III

To compare the views of families living with SMA towards PCGS/PNGS screening with the views of people with no prior experience of SMA, the SMA Screening Survey (UK) questions on PCGS/PNGS were replicated to produce a shorter more focused survey (the UK GenPop Survey). Key questions were replicated to allow direct comparison of the data generated from both surveys. As the UK general population are understood to have poor knowledge of SMA,^28^ information was included within the UK GenPop Survey to explain what SMA is and how it affects people in its various forms. This information was replicated with permission from SMA Support UK's resources and as such is certified by the UK Information Standard.

To access people with no prior experience of SMA, The UK GenPop Survey was distributed digitally through the School of Life Sciences (University of Warwick), including students and staff. Participants were then asked to circulate the survey through social media (Facebook). Unlike the SMA Screening Survey (UK), the UK GenPop Survey was only available in online format. To reduce the impact of selection bias and over‐representation of students, participants <25 years of age were excluded from the analysis.

### 2.4 ill‐equipped User involvement

As well as the underpinning qualitative work, the three phases of the research were also supported by expert review panels. The first of these panels comprised of six staff members from SMA Support UK. The second group consisted of four members of SMA Patient Registry staff. These two professional panels reviewed the SMA Screening Survey (UK) once it had been completed in its draft form, offering feedback on the questions as well as advice on the implementation strategy. A separate expert review panel, made up of people living with SMA (nine people who had SMA themselves and six who had a relative with SMA), met to discuss the phase I qualitative analysis, the early design of the survey as well as to offer feedback on the first completed draft of the SMA Screening Survey (UK). A final meeting of this panel was held upon completion of the quantitative survey analysis to discuss dissemination and the wider implications of the findings.

### 2.5 Data stratification and statistical analysis

Responses to each question were stratified as follows: gender (Male 1 *v* Female 0); age (35‐45 1 *v* other 0); qualifications (degree or above (1) *v* other (0)); religious (yes (1) *v* no (0)); do you have children (yes (1) *v* no (0)); relationship to SMA (patient (1) *v* family (0)); type of SMA associated with your family (type 0 or type 1(1) *v* other (0)); living or lived with an SMA patient (yes (1) *v* no (0)); current health (good or very good (1) *v* other (0)); current pregnant or trying to get pregnant (yes (1) *v* no (0)). For all questions regarding screening answers were stratified as either agree/strongly agree (1) or other (0). This was done because it allowed the simplest way of assessing the positive views of respondents.

The attitudes of the general population towards PCGS/PNGS SMA screening were compared to responses from responses from the SMA‐associated population (n=337) to identify major differences. These were then further investigated by comparing the general population with: 1) adults with SMA (types II and III); and 2) family members associated with SMA (types I, II and III) to determine if there were any statistical differences (N.B. due to the poor prognosis associated with SMA type I, no adults with this diagnosis were included in the study). For each question the number of “agree” vs “other” responses were reported and statistical differences between the subgroups were assessed using a chi‐squared analysis (GraphPad Prism software, v6). It is important to note that for the subanalysis, the SMA‐associated sample was reduced to 287 participants through the removal of people associated with variant types of SMA (type IV SMA, Spinal Bulbar Muscular Atrophy and Spinal Muscular Atrophy with Respiratory Distress). This was done to enable a more meaningful comparison with the general population data.

## 3 RESULTS

### 3.1 Non‐SMA *v* SMA populations: Comparative cohort descriptive characteristics

In total, there were 336 responses from the general population and 337 from SMA‐associated families (255 family members (75.7%) and 82 (24.3%) with SMA themselves). Basic descriptive analysis of the general population data revealed that 146 (43%) were female; 187 (56%) were educated to degree level or higher and 208 (62%) were religious (Table 1). When these values were compared to the demographics of SMA‐associated participants, there were significant differences in the gender, with a higher proportion (75%) of the SMA‐associated participants being female (*P*<.0001) and less highly educated, with 64% of the cohort not educated to degree level (*P*<.0001) (Table 1). Levels of religiosity, however, appeared similar between the two groups (*P*=.96; Table 1).

**Table 1:** Characteristics and demographics of survey responders. Demographics are shown for responders from the general population (n=336), responders associated with SMA families (n=337). Response distributions were compared between the two groups and significant differences were assessed using chi‐squared analysis (*P*‐value)

Characteristic	General population (n=336)	SMA screening survey (n=337)	*P*‐Value^a^
Gender ‐ no. (%)			<.0001
Male	190 (57%)	85 (25%)	
Female	146 (43%)	251 (75%)	
Age			<.0001
18‐25 y	Excluded^b^	16 (5%)	
26‐34 y	79 (24%)	40 (12%)	
35‐45 y	80 (24%)	99 (29%)	
46‐55 y	108 (32%)	76 (23%)	
56‐65 y	44 (13%)	49 (15%)	
>65 y	25 (7%)	56 (17%)	
Qualifications			<.0001
Degree or higher	187 (56%)	122 (36%)	
Other/none	149 (44%)	215 (64%)	
Religious			.96
Yes	208 (62%)	185 (55%)	
No	128 (38%)	113 (45%)	

a *P* values were calculated with the use of the chi‐squared test.

b 18‐ to 25‐y‐olds were excluded to reduce selection bias (see Section 2).

### 3.2 Non‐SMA *v* SMA populations: Comparisons of views on pre‐conception genetic screening (PCGS)

A direct comparisons on the two studied populations (non‐SMA and SMA) shows a significant difference in the levels of support for PCGS (86% *v* 77%, respectively; *P*=.004) (Table 2). This difference is predominantly driven by participants associated with type II SMA. Indeed, the levels of support in the general population (86%) were similar to the support expressed by people associated with type I (family members 88%, *P*=.47) and type III (Family members 73%, *P*=.09; adults with SMA 94%, *P*=.22). However, significantly less support was observed in both family members associated with type II SMA (72%, *P*=.003) and adults with type II SMA (63%, *P*=.001) (Table 1).

**Table 2:** Response summaries for questions assessing views on pre‐conception genetic screening (PCGS). Response breakdowns are shown for the general population, SMA‐associated family subgroups (type I, type II and type III) and adults with SMA (type II and type III). Responses for each question were stratified as “agree” *v* “other” (other= disagree and neither disagree nor agree). Response distributions were compared using chi‐squared analysis (*P*‐value; significant differences are highlighted (*P*<.05)

		SMA families				Adults with SMA		Statistical comparison (chi‐squared analysis)					
	Non‐SMA Population (n=336)	SMA Population (n=337)	Type IF (n=120)	Type II F (n=87)	Type III F (n=22)	Type II AwS (n=27)	Type III AwS (n=31)	Non‐SMA *v* SMA	Non‐SMA *v* Type I (F)	Non‐SMA *v* Type II (F	Non‐SMA *v* Type III (F)	Non‐SMA *v* Type II (AwS)	Non‐SMA *v* Type III (AwS)
I would support a pre‐conception genetic screen for SMA								0.004	0.47	0.003	0.09	0.001	0.22
Agree	288 (86%)	260 (77%)	106 (88%)	63 (72%)	16 (73%)	17 (63%)	29 (94%)						
Other	48 (14%)	77 (23%)	14 (12%)	24 (28%)	6 (27%)	10 (37%)	2 (6%)						
Identifying SMA carriers before pregnancy will reduce the number if SMA‐associated terminations								0.03	0.98	0.01	0.32	0.003	0.93
Agree	269 (80%)	247 (73%)	96 (80%)	59 (68%)	17 (77%)	15 (56%)	25 (80%)						
Other	67 (20%)	90 (27%)	24 (20%)	28 (32%)	5 (23%)	12 (44%)	6 (20%)						
Idenitfying SMA carriers in the general population will increase awareness of SMA as a conditon								0.22	0.06	0.009	0.52	0.008	0.57
Agree	304 (90%)	295 (88%)	115 (96%)	70 (80%)	19 (86%)	20 (74%)	29 (94%)						
Other	32 (10%)	42 (12%)	5 (4%)	17 (20%)	3 (14%)	7 (26%)	2 (6%)						
Pre‐conception screening is a form of social engineering								0.01	0.28	0.06	0.31	<0.0001	0.15
Agree	62 (18%)	88 (26%)	17 (14%)	24 (28%)	6 (27%)	15 (56%)	9 (29%)						
Other	274 (82%)	249 (74%)	103 (86%)	63 (72%)	16 (73%)	12 (44%)	22 (71%)						
Idenitfying SMA carriers in the general population will lead to carriers feeling stigmatised								0.11	<0.0001	0.35	0.68	0.02	0.51
Agree	94 (28%)	76 (26%)	10 (8%)	20 (23%)	7 (32%)	13 (48%)	10 (32%)						
Other	242 (72%)	261 (74%)	110 (92%)	67 (77%)	15 (68%)	14 (52%)	21 (68%)						
Identifying SMA carriers before pregnancy would affect people's choice of reproductive partners								0.01	0.42	0.09	0.02	0.08	0.003
Agree	118 (35%)	151 (45%)	47 (39%)	39 (45%)	13 (59%)	14 (52%)	19 (61%)						
Other	218 (65%)	186 (55%)	73 (61%)	48 (55%)	9 (41%)	13 (48%)	12 (39%)						

### 3.3 SMA subtypes *v* general population: Reasons for and against support of pre‐conception genetic screening

Assessment of the PCGS subquestions indicate that the majority of the general population thought PCGS was beneficial because they believed it would reduce the number of SMA‐associated terminations (80%; Table 2). In comparison, a much lower percentage of family members and adults with type II SMA thought PCGS would reduce the number of terminations (GP 80% *v* families 68%, *P*=.01; *v* adults with type II 56%, *P*=.003; Table 2). The majority of participants from the general population also thought that PCGS would raise awareness of SMA in the general population (GP 90%). This belief was also reflected in the responses of SMA‐associated participants (88%; Table 2) with the exception of those participants associated with type II SMA (type II family members 80%, *P*=.009; adults with type II 74%, *P*=.008) (Table 2). Moreover, adults with type II SMA were significantly more likely than any other group included in this analysis to believe that PCGS is a form of social engineering (GP 18% *v* adults with type II 56%, *P*<.0001; Table 2).

While overall, type I associated participants were the group most similar to the general population than any other SMA‐associated participants, differences between these two groups also emerged: more participants in the general population thought that PCGS would lead to carrier stigmatization (GP 28% *v* adults with type II 8%, *P*<.0001; Table 2) and more type I family members thought it would raise SMA awareness (type I family members 90% *v* GP 96%, *P*=.06; Table 2).

Only one significant difference was identified between the general population and type III participants, with fewer participants from the general population agreeing that carrier identification would change their choice of reproductive partners (GP 35% *v* type III families 59%, *P*=.02; *v* adults with type III 52%, *P*=.003; Table 2).

### 3.4 Non‐SMA *v* SMA populations: Comparison of views on prenatal genetic screening (PNGS)

Overall, 84% of non‐SMA‐associated participants surveyed were in favour of PNGS (Table 3). As with PCGS, this was significantly higher than amongst the SMA‐associated population (76%; *P*=.009) (Table 3). Again, this difference was predominantly driven by participants associated with type II SMA (family members 72%, *P*=.005; adults with type II 52%, *P*=.002; Table 3).

**Table 3:** Response summaries for questions assessing views on prenatal genetic screening (PNGS). Response breakdowns are shown for the general population, SMA‐associated family subgroups (type I, type II and type III) and adults with SMA (type II and type III). Responses for each question were stratified as “agree” *v* “other” (other= disagree and neither disagree nor agree). Response distributions were compared using chi‐squared analysis (*P*‐value; significant differences are highlighted (*P*<.05)

		SMA Families				Adults with SMA		Statistical comparison (chi‐SMA Families squared analysis)					
	Non‐SMA Population (n=336)	SMA Population (n=337)	Type I F (n=120)	Type II F (n=87)	Type III F (n=22)	Type II AwS (n=27)	Type III AwS (n=31)	Non‐SMA *v* SMA	Non‐SMA *v* Type I (F)	Non‐SMA *v* Type II (F)	Non‐SMA *v* Type III (F)	Non‐SMA *v* Type II (AwS)	Non‐SMA *v* Type III (AwS)
I would support a prenatal screening programme for SMA								0.009	0.38	0.005	0.06	0.002	0.61
Agree	283 (84%)	257 (76%)	105 (88%)	62 (72%)	15 (68%)	14 (52%)	25 (81%)						
Other	53 (16%)	80 (24%)	15 (12%)	25 (29%)	7 (32%)	13 (48%)	6 (19%)						
Screening for SMA in pregnancy would enable everyone to make informed decision								0.54	0.21	0.91	0.36	0.06	0.92
Agree	284 (85%)	279 (83%)	107 (89%)	70 (80%)	17 (77%)	19 (70%)	26 (84%)						
Other	52 (15%)	58 (17%)	13 (11%)	17 (20%)	5 (23%)	8 (30%)	5 (16%)						
Screening for SMA in pregnancy will prevent unnecessary suffering								<0.0001	0.007	0.01	0.51	<0.0001	0.21
Agree	222 (66%)	201 (60%)	95 (79%)	45 (52%)	13 (59%)	6 (22%)	17 (55%)						
Other	114 (34%)	136 (40%)	25 (21%)	42 (48%)	9 (41%)	21 (78%)	14 (45%)						
Screening for SMA in pregnancy will raise awareness of the condition in the general population								0.19	0.57	0.28	0.18	0.01	0.61
Agree	293 (87%)	282 (84%)	107 (89%)	72 (83%)	17 (77%)	19 (70%)	28 (90%)						
Other	43 (13%)	55 (16%)	13 (11%)	15 (17%)	5 (23%)	8 (30%)	3 (10%)						
Idenitfying SMA carriers in the general population will increase awareness of SMA as a conditon								<0.0001	<0.0001	<0.0001	0.01	<0.0001	<0.0001
Agree	74 (22%)	180 (53%)	49 (41%)	57 (66%)	10 (45%)	19 (70%)	18 (58%)						
Other	262 (78%)	157 (47%)	71 (59%)	30 (34%)	12 (55%)	8 (30%)	13 (42%)						
It would be a loss to society to have fewer people with SMA coming into the world								<0.0001	0.01	<0.0001	0.52	<0.0001	0.005
Agree	32 (10%)	93 (28%)	22 (18%)	28 (32%)	3 (14%)	18 (67%)	8 (26%)						
Other	304 (90%)	244 (72%)	98 (82%)	59 (68%)	19 (86%)	9 (33%)	23 (74%)						
It would be difficult for pregnant couples to refuse screening for SMA during pregnancy								<0.0001	<0.0001	0.0006	0.004	0.11	0.004
Agree	166 (49%)	86 (26%)	27 (23%)	25 (29%)	4 (18%)	9 (33%)	7 (23%)						
Other	170 (51%)	251 (74%)	93 (77%)	62 (72%)	18 (82%)	18 (67%)	24 (77%)						
Screening for SMA in pregnancy is useful evening if the Type of SMA can not be determined								0.0006	<0.0001	0.4	0.21	0.31	0.08
Agree	184 (55%)	228 (68%)	92 (77%)	52 (60%)	15 (68%)	12 (44%)	22 (71%)						
Other	152 (45%)	109 (32%)	28 (23%)	35 (40%)	7 (32%)	15 (56%)	9 (29%)						
Termination of milder forms of SMA is unfortunately necessary to reduce the number of severe SMA children being born								0.37	0.02	0.15	0.76	0.04	0.69
Agree	97 (29%)	108 (32%)	48 (40%)	32 (37%)	7 (32%)	3 (11%)	10 (32%)						
Other	239 (71%)	229 (68%)	72 (60%)	55 (63%)	15 (68%)	24 (89%)	21 (68%)						

### 3.5 SMA subtypes *v* general population: Reasons for and against support of pre‐conception genetic screening (PCGS)

The majority of the general population agreed that PNGS would allow everyone to make informed decisions (85%) and this belief was widely held by all analysed participants (Table 3). The general population generally agreed that PNGS would prevent suffering (66%). In comparison, however, significantly fewer type II associated participants agreed with this statement (GP, 66%; type II families 52%, *P*=.01; adults with type II 22%, *P*<.0001) (Table 3). In contrast, significantly more type I families thought it would reduce suffering (79%, *P*=.007; Table 3). Non‐SMA‐associated participants generally agreed that a PNGS programme would raise awareness of SMA in the general population (87%) (Table 3). While most SMA‐associated groups agreed with this, there was significantly less agreement amongst adults with type II SMA (GP (87%) *v* type II adults (70%; *P*=.01; Table 3).

It is noteworthy that only 22% of participants from the general population thought that people diagnosed with SMA could live fulfilling lives; this was significantly lower than all SMA‐associated participants, even those associated with type I SMA, whose views elsewhere have been more closely aligned with the general population (Table 3). This finding was also underscored by the fact that only 10% of the general population agreed that a reduction in the number of SMA children coming into the world would be a loss to society—this was significantly lower than all SMA‐associated subgroups with the exception of type III family members (14%; Table 3).

The possible inability of members of the public to refuse the screen emerged as a more significant concern to members of the general population than it was to all SMA‐associated participants except for adults with type II SMA (type I family members 49% *v* 23%, *P*<.0001; type II family members 49% *v* 29%, *P*=.0006; type III family members 49% *v* 18%, *P*=.004; adults with type III SMA 49% *v* 23%, *P*=.004) (Table 3). Indeed, this concern around ability to refuse screening was the only question for which the views of non‐SMA‐associated participants most closely aligned with those of adults with type II SMA.

While the views of type I families and the general population were relatively closely matched on questions regarding PCGS, areas of divergence most clearly emerged within the PNGS subquestions. One of these concerned the ability of genetic technologies to provide information on type. Thus far, the lack of accurate information on disease severity that could be provided through PNGS for SMA has been regarded as a major stumbling block to its introduction. For families living with type I SMA, however, unlike the general population this was not considered a preclusion to its introduction, with significantly more agreeing that screening is still useful even with these limitations (type I family members 77% *v* GP 55%; *P*<.0001) (Table 3). Moreover, when compared to the general population, significantly more type I family members than any other SMA‐associated group agreed that termination of foetuses with milder forms of SMA is an unfortunate (but necessary) by‐product if we are to ensure that children with severe SMA are not born (type I families 40% *v* GP 29%, *P*=.02) (Table 3). In contrast, the group expressing the strongest opposition to this notion was adults with type II SMA (type II adults 29% *v* GP 11%, *P*=.04) (Table 3), with over double the number of members of the general public agreeing with the statement than type II adults.

## 4 DISCUSSION

This paper presents the first extended comparison of the views of the general population and families associated with SMA on potential PCGS and PNGS programmes in the UK. The data presented here reveal that both groups are generally in favour of both screening programmes, although PCGS elicited slightly more supported than PNGS. This is in keeping with previous reports^28^, ^29^ and reflects a widespread belief in the importance of earlier screens and the prevention of SMA without the need for selective pregnancy termination. Overall, however, the population without prior experience of SMA was more supportive of both types of screening than SMA‐associated participants.

The major area of divergence between the two studied populations appeared to centre around the way life with SMA was valued. Indeed, the general population viewed SMA significantly more negatively than SMA‐associated families, the only exception being type I families, whose views were more closely aligned to those of the general population. This correlation suggests that when considering screening decisions, and in light of the inability of genetic technologies to accurately discern SMA type that people lacking prior knowledge of SMA may imagine screening decisions in terms of the “worst case scenario” and wish to guard against such an eventuality. In contrast, people with experience of SMA appeared to imagine future children with SMA within the framework of their existing experiential knowledge; it was challenging for them to imagine such future lives as involving anything other than a repetition of that with which they were familiar.^30^ This is in spite of the fact that it is possible for children with different types of SMA to be born to the same carrier parents. This finding highlights that it is not only possession of experiential knowledge, but also the *nature* of that experiential knowledge that is critical in the formulation of screening attitudes.

The significance of the nature of SMA experience also appeared in the subanalysis of type II adults and families. Participants associated with this type of SMA were more likely than any other group to reject carrier screening programmes and to view the associated decline in numbers of people with SMA in the world as a loss to society. That adults with type II SMA are a group distinct from other adults diagnosed with SMA is an observation noted elsewhere in the literature.^31^, ^32^ Kruitwagen‐Van Reenen et al.,^32^ for example, argue that adults with type II SMA, despite their more severe clinical presentation than adults with type III, were more likely to rate their quality of life as high. We have argued elsewhere that this finding also emerged within our own dataset and have argued that this may be due to the relatively fixed and congenital nature of type II impairment^8^ when compared to the degenerative effects associated with both types I and III. Adults with type II, having always had their disability, are often well adjusted to it and develop their plans for their lives around its existence. Adults with types III SMA, however, while experiencing clinically milder symptoms, often go through periods of decline or deterioration in abilities and many eventually come to rely on a wheelchair. These contrasting experiences of SMA were inextricably bound up with the way in which adults with SMA related to and perceived their condition, as well as its ramifications in terms of personal identity.^8^ Adults with type II SMA more frequently spoke of their condition being an integral (and highly valued) part of their sense of self, a finding not nearly as prevalent amongst adults with milder disease. This finding is also reflected within disability studies as well as within the rehabilitation literature exploring attitudes to cure,^33^, ^34^ whereby contrasting perspectives are observed between people who have relatively static congenital impairments (eg deafness), and those who acquire their impairment suddenly and unexpectedly (eg spinal cord injury), or who experience decline over time (eg Motor Neurone Disease).

While the nature (in terms of disease severity) of a person's experiential knowledge of SMA (or lack of) was found to be critical to the formulation of screening perspectives, it is also noteworthy that whether or not the SMA‐associated participant had SMA themselves (or were a family member of someone with SMA), did not significantly impact on reproductive attitudes. It has been argued that people with genetic diseases are the “best experts” on their own condition—that having the condition oneself affords that person a privileged vantage point from which to imagine the implications of having a child with that same condition.^35^, ^36^ Our present analysis, however, highlights that “empathetic” forms of experiential knowledge (ie knowing SMA through the experiences of a family member) could be just as influential in determining attitudes towards screening as embodied forms (having the condition oneself).^8^ The centrality of empathetic knowledge to reproductive attitudes and decisions has been highlighted elsewhere.^10^, ^37^, ^38^ This present study suggests that it may be equally as important to incorporate the views of people with empathetic forms of experiential knowledge when exploring the reproductive attitudes of people affected by genetic diseases. Indeed, despite not having the condition themselves, the viewpoints of family members were shown by this study to be more similar to those of adults with SMA (with the same type) than they were to the general population, highlighting the pervasiveness of both embodied and empathetic experience in altering attitudes.

Further research is indicated to explore perceptions of disease severity in the context of genetic screening programmes across a wider spectrum of genetic diseases and incorporating different symptoms not typically associated with SMA (eg cognitive impairment, pain). As this study highlights, the correlation between disease severity and support for genetic screening programmes was not straightforward; screening support could not be accurately predicted based on the severity of SMA a person had experience of. This dislocation of the condition's (clinical) severity from the various ways it came to be experienced and valued in both the contexts of everyday life, but also in the arena of reproduction, warrants further and serious consideration. As the capacities of reprogenetic technologies continue to expand and increasing numbers of conditions fall within its remit, policy decisions need to be made concerning which conditions should be included on genetic screening panels (and offered to the general public) and which should not. While the clinical severity of the disease may be amongst the most important considerations for such decisions (particulary in the context of a publicly funded health‐care system), this study highlights the critical dissonance between the clinical presentation of a condition, and a person's lived experience of it.

## 5 CONCLUSIONS

Overall, this study highlights that while support for PNGS and PCGS was high amongst both SMA‐associated participants and the general population, that clear differences nevertheless emerged between the two populations, with the general public generally taking a far more negative view of SMA than the families who live with it. This was with the exception of type I families, whose responses more closely mirrored those of the general population than other SMA‐associated participants. In so doing, this study highlights the centrality of experiential knowledge in determining reprogenetic attitudes, and consequently its significance to future genetic screening programmes, both in terms of policy debates, but also in terms of information provision, for prospective parents facing increasingly abstract‐ and increasingly complex‐reproductive decisions.

### 5.1 Limitations

Due to confidentiality and data protection issues, no identifiable data were asked of individuals who participated in the SMA Screening Survey (UK) or the UK GenPop Survey, including IP addresses (where the survey was completed online). This meant that there was no mechanism in place to prevent an individual completing multiple surveys. Due to the poor prognosis associated with type I SMA, all of the adults with SMA who participated in this study were diagnosed with clinically less severe forms of SMA (types II and III). While this may have influenced the way in which SMA was perceived, we feel this limitation was minimized by the inclusion of type I associated family members. Furthermore, all forms of SMA can be associated with significant disability. Participants who completed the UK Genpop Survey were recruited through the University of Warwick's School of Life Sciences and through social media (Facebook links to the survey). It is possible that this recruitment strategy introduced bias in the sample. Efforts were made to circumvent this limitation, however, by excluding all Genpop participants under the age of 25 from the analysis (130 in total).

## CONFLICT OF INTEREST

The authors have no conflicting interests.
